# Reflux esophagitis patients exhibit reduced gastric tolerance, impaired slow-wave activity and esophageal dysmotility

**DOI:** 10.3389/fmed.2026.1897880

**Published:** 2026-08-12

**Authors:** Yi Yang, Xueying Yin, Xiaoyan Wang, Jing Zhang, Jun Yu, Fengming Yue, Lixia Wang, Dong Yang, Zhifeng Zhang, Xiaoyu Sun, Xiukun Hou, Zhijun Duan

**Affiliations:** 1 The Second Department of Gastroenterology, The First Affiliated Hospital of Dalian Medical University, Dalian, China; 2 The Third Department of Gastroenterology, Affiliated Central Hospital of Dalian University of Technology, Dalian, China; 3 The First Ultrasound Department, The First Affiliated Hospital of Dalian Medical University, Dalian, China; 4 The First Department of Gastroenterology, Xinhua Hospital Affiliated to Dalian University, Dalian, China

**Keywords:** esophageal motility, gastric motility, multi-dimensional evaluation, reflux esophagitis, visceral hypersensitivity

## Abstract

**Background:**

Reflux esophagitis (RE) is an endoscopic subtype of gastroesophageal reflux disease (GERD). Although gastric dysfunction is known to contribute to GERD, a comprehensive assessment of gastric motor function specifically in RE patients remains lacking.

**Objective:**

To comprehensively evaluate gastric motor function in patients with reflux esophagitis (RE) using a multidimensional non-invasive protocol, and to explore the associations between gastric motility abnormalities and esophageal dysmotility.

**Methods:**

Forty RE patients diagnosed by endoscopy and high-resolution manometry (HRM) and 34 healthy controls were enrolled between April 2019 and January 2020 based on feasibility of recruitment during the study period. Gastric tolerance and visceral sensitivity were evaluated using a nutrient drink test combined with a visual analog scale (VAS). Gastric emptying was assessed by gastrointestinal ultrasound, and gastric myoelectrical activity was recorded by electrogastrography (EGG). Esophageal motility was simultaneously evaluated by HRM.

**Results:**

Compared with controls, RE patients showed significantly reduced maximum tolerated volume (MTV), higher postprandial VAS scores for fullness, nausea and pain, lower gastric emptying rates at 20 and 30 min postprandially, and a lower percentage of normal postprandial slow waves (all *p* < 0.05). After normalization to ingested volume (symptom load index), RE patients still exhibited a significantly higher symptom load for fullness and pain at all postprandial time points, and for nausea at 10, 20, and 30 min (all *p* < 0.01). Heartburn severity was positively correlated with pain score at 0 min postprandial (*r* = 0.442, *p* = 0.004), and regurgitation severity was positively correlated with gastric emptying rate at 30 min (*r* = 0.512, *p* = 0.001). The 20-min fullness score was negatively correlated with distal contractile integral (DCI) (*r* = −0.353, *p* = 0.025).

**Conclusion:**

RE patients exhibit multiple gastric motility abnormalities, including reduced gastric tolerance, visceral hypersensitivity, delayed gastric emptying, and postprandial gastric dysrhythmia. These gastric dysfunctions are associated with typical RE symptoms and selectively correlate with esophageal contractile indices, suggesting that RE may represent a disorder of upper gastrointestinal motility.

## Introduction

The digestive system condition known as gastroesophageal reflux disease (GERD) is typified by the reflux of stomach contents into the esophagus, which can cause discomfort and/or histological issues (1). Globally, the overall prevalence of GERD is approximately 14% (2). Although the prevalence in Asia is significantly lower than in North America and European countries, it has shown a noticeable upward trend in recent years (3). The disease not only imposes a heavy dual burden on society in terms of economic costs and health impacts (4), but also carries a potential risk of inducing related tumors (5). It has now become one of the common digestive system diseases threatening human health. Non-erosive reflux disease (NERD), reflux esophagitis (RE), and Barrett’s esophagus (BE) are the three primary clinical phenotypes of GERD based on endoscopic and histological findings (4).

The pathogenesis of GERD is complex, with the core being an imbalance between the esophageal defense mechanism and the invasive effect of reflux substances. Since the mid-20th century, the role of esophageal motility dysfunction in GERD has received increasing attention. Current studies consistently show that the incidence of abnormal esophageal motility in GERD patients is relatively high; weakened or absent peristaltic function will delay the clearance of reflux substances, thereby exacerbating mucosal damage (6, 7). However, the pathophysiological process of GERD may not be limited to the esophagus. Clinically, patients with RE often present with upper gastrointestinal symptoms, such as early satiety and post-meal abdominal distension, which highlights the crucial role of the gastric function status. Studies have confirmed that abnormal gastric motility is very common in GERD patients (8–10). GERD patients with food regurgitation exhibit gastric dysrhythmias, and 50% of subjects demonstrated delayed emptying of either the solid or liquid component of the stomach, or slow liquid emptying (11). However, there is currently a lack of systematic assessment of gastric function specifically in RE patients. Given that traditional assessment methods are invasive or involve radiation exposure, there is a significant clinical need to develop and apply a simple and non-invasive assessment approach for evaluating gastric function in GERD patients.

We therefore hypothesized that the pathogenesis of reflux esophagitis (RE) involves not only local esophageal dysmotility but also gastric motility abnormalities, such as impaired gastric accommodation and abnormal gastric slow-wave activity. These gastric dysfunctions may increase intragastric pressure, promote reflux events, and act synergistically with esophageal dysmotility to form a comprehensive upper gastrointestinal motor disorder background in RE. To test this hypothesis, drawing on our research group’s previous successful experience in the multidimensional assessment of functional dyspepsia (FD) (12). The present study aimed to: (1) comprehensively evaluate gastric motor function (using non-invasive electrogastrography and gastrointestinal ultrasound) and esophageal motor function (using high-resolution manometry) in patients with RE; (2) characterize the patterns of gastric and esophageal motility abnormalities in RE patients; and (3) explore the correlation between gastric and esophageal dysmotility. The findings may provide new pathophysiological insights into RE and support the development of personalized treatment strategies targeting integrated gastroduodenal and esophageal motility.

## Methods

### Study participants

**Figure 1:**
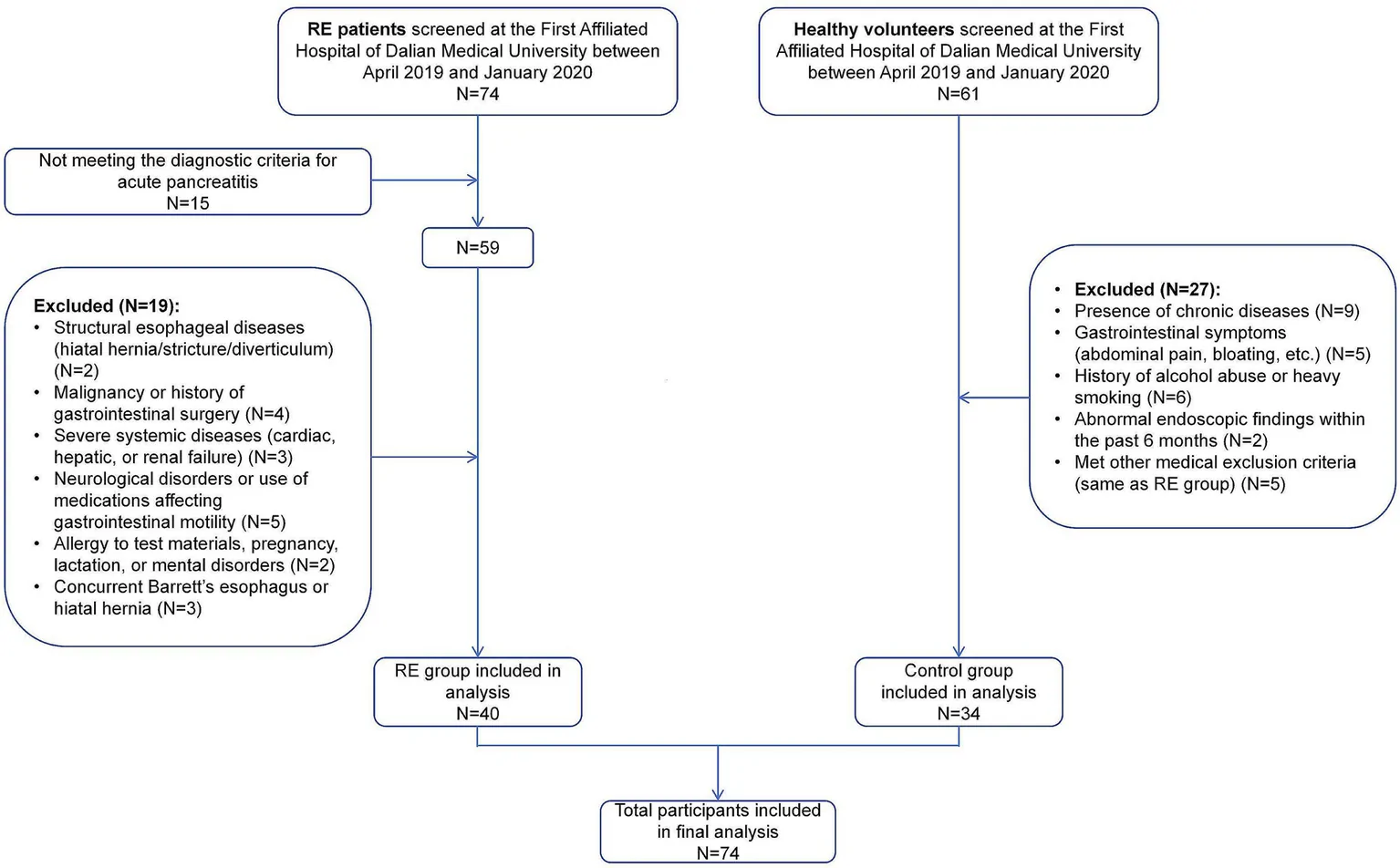
Flow diagram of subject enrollment in this case–control study.

This was a case–control study that enrolled 40 patients diagnosed with reflux esophagitis (RE) and meeting the inclusion and exclusion criteria from the First Affiliated Hospital of Dalian Medical University between April 2019 and January 2020. Concurrently, 34 healthy volunteers without chronic diseases or relevant clinical symptoms were recruited as the control group (Figure 1). The study was conducted in accordance with the principles of the Declaration of Helsinki. The rights and privacy of all participants were fully protected. The study protocol was approved by the Ethics Committee of Dalian Medical University (Approval No. PJ-KS-KY-2019-37) and was registered with the Chinese Clinical Trial Registry (Registration No. ChiCTR1900026457).

Inclusion Criteria of RE group: (1) Age ≥18 years; (2) presence of symptoms such as regurgitation, heartburn, or retrosternal pain; and (3) endoscopic findings indicative of reflux esophagitis (13).

Inclusion Criteria of control group: (1) No endoscopic evidence of space-occupying lesions, hiatal hernia, or esophageal mucosal injury within the 6 months prior to enrollment; (2) absence of any chronic diseases; (3) lack of clinical symptoms related to the gastrointestinal tract, such as diarrhea, constipation, nausea, vomiting, bloating, acid reflux, or abdominal discomfort; and (4) no history of severe smoking or alcohol misuse.

Exclusion Criteria: (1) Patients with structural esophageal diseases such as hiatal hernia, esophageal stricture, or esophageal diverticulum; (2) patients with malignant tumors; (3) patients with a history of gastrointestinal surgery; (4) patients with severe systemic diseases including, but not limited to, uncontrolled cardiovascular, hepatic, or renal insufficiency; (5) patients with neurological disorders or taking medications known to affect gastrointestinal motility; (6) allergy to electrodes or the nutrient meal; (7) pregnant or lactating women; (8) individuals with unstable mental health conditions or cognitive dysfunction; and (9) patients with concurrent conditions such as Barrett’s esophagus or hiatal hernia.

### Methods

To minimize inter-operator variability, all subjects were conducted by the same researcher. Additionally, all of the following tests were conducted by the same examiner at the same location.

#### Baseline data

Collect baseline data for all study subjects, including age, sex, height, and weight, and calculate the body mass index (BMI) based on these measurements.

#### Gastroesophageal reflux disease questionnaire

The existence and severity of gastroesophageal reflux symptoms were thoroughly evaluated using the GERDQ in conjunction with endoscopic findings. Heartburn, regurgitation, epigastric discomfort, nausea, sleep disturbance, and the use of over-the-counter drugs were among the upper gastrointestinal symptoms that were retrospectively assessed using the GERDQ during the previous week (14). A GERDQ score of ≥8 was considered indicative of GERD, whereas a score of <8 was considered negative for GERD, according to the widely used diagnostic cutoff (14).

#### Nutrient meal tolerance test

A modified nutrient meal test was conducted to evaluate gastric tolerance. Specifically, 100 grams of milk powder (Nestlé) and 50 grams of Milo (Nestlé) were dissolved in 1200 mL of boiling water. The mixture was stirred uniformly and cooled to 37 °C to prepare the test nutrient solution. Participants drank the solution at a constant rate of 60 mL/min until their satiety score on a visual analog scale reached 100. The total volume of liquid ingested at the point of cessation was recorded as the maximum tolerated volume (MTV) (15, 16). The MTV reflects a composite of gastric accommodation, gastric wall tension, and visceral sensitivity.

#### Symptom assessment using visual analog scale

A 100-mm horizontal visual analog scale (VAS) was used to quantify postprandial symptoms of fullness, nausea, and pain at 0, 10, 20, and 30 min after the meal to reflect changes in visceral sensitivity (16). To account for the potential circularity between VAS assessment and MTV determination, a symptom load index was calculated as (VAS score / MTV) × 100, representing the symptom intensity per 100 mL of nutrient solution consumed. This index was used to normalize postprandial symptom severity to the actual ingested volume.

#### Gastrointestinal ultrasound for gastric emptying assessment

Gastric emptying was assessed using a Philips HD15 ultrasound system (USA) equipped with a convex array probe operating at a frequency of 3.5–5.0 MHz. After fasting for 8 h, participants ingested 450 mL of gastrointestinal ultrasound contrast agent (manufacturer: East Asia Pharmaceuticals, Huzhou; product standard: YZB China 0058-2010) in a single continuous intake. Using the Bolondi method, the antral area was measured during both the diastolic and contraction phases at 5, 10, 15, 20, and 30 min post-ingestion. The system automatically calculated the diastolic area at each time point. The gastric emptying rate (GER%) was calculated using the formula: GER% = (Change in Diastolic Area/Initial Diastolic Area) × 100%, with the diastolic area at 5 min post-ingestion serving as the baseline, to evaluate gastric emptying function (17).

#### Electrogastrography

A multi-channel gastrointestinal electrical recorder (MEG-04A, Medda Medical Equipment Co., Ltd., Ningbo, China) was used to record gastric myoelectrical activity in accordance with the modified Chen Jiande method (18). Thirty minutes prior to and thirty minutes following the meal, recordings were made. Two active electrodes were placed on the abdominal surface over the gastric antrum and corpus, with a reference electrode positioned on the right costal margin. The skin was abraded and cleaned with alcohol to reduce impedance below 5 kΩ. Gastric myoelectrical activity was recorded in the supine position after a 30-min rest. The signal was acquired at a sampling rate of 100 Hz, band-pass filtered between 0.016 Hz and 0.25 Hz (corresponding to 1–15 cycles per minute), and analyzed using fast Fourier transformation. The dominant frequency (DF), dominant power (DP), and the percentage of normal slow waves (2.4–3.7 cpm) were calculated for both preprandial (30 min) and postprandial (30 min) recordings.

#### High-resolution esophageal manometry

Esophageal motor function was assessed using a high-resolution esophageal manometry catheter system with GAP-36A data acquisition software (meddynamics Incorporated, Medda Medical Equipment Co., Ltd., Ningbo, China). After fasting for at least 8 h, the manometry catheter was transnasally placed into the stomach. The catheter position was adjusted and stabilized for 3–5 min. Following stabilization of the upper esophageal sphincter (UES) and lower esophageal sphincter (LES) pressures, resting pressures were recorded for at least 30 s. Subsequently, participants performed up to 10 swallow tests, each involving a 5 mL bolus of room-temperature water, with a 30-s interval between swallows. Repeated swallowing after a single bolus was not permitted. All data were analyzed using Manoview 3.0 software and interpreted according to the Chicago Classification v3.0 criteria (19).

#### Hospital anxiety and depression scale

The HADS was used to assess the psychological status of both the RE group and the healthy control group. The self-administered questionnaire HADS was used to evaluate anxiety and depression in individuals who are not psychiatric. It has fourteen items, seven of which measure anxiety (HADS-A) and seven of which measure depression (HADS-D) (20).

### Statistical analysis

SPSS version 27.0 was used for all statistical analyses. GraphPad Prism 9.5 was used to visualize the data. The independent samples *t*-test was used to compare normally distributed continuous data, which were reported as mean ± SD. The Mann–Whitney *U* test was used to compare non-normally distributed continuous data, which were represented as *M* (*Q_25_*, *Q_75_*). Fisher’s exact test or the chi-square test were used to compare categorical data that were displayed as *n* (%). Statistical significance was defined as a *p*-value of less than 0.05.

## Results

### Demographic characteristics

A total of 40 patients with RE and 34 healthy controls were enrolled in this study. There were no significant differences between the two groups in age, sex, height, or BMI (*p* > 0.05). The total GERD-Q score and all individual item scores were significantly higher in the RE group than in the control group (*p* < 0.05), with heartburn and regurgitation being the most common symptoms in RE patients. Additionally, the RE group’s HADS-A and HADS-D scores were considerably greater than those of the healthy controls (*p* < 0.05, Table 1).

**Table 1:** Baseline characteristics of the control and RE group.

Baseline data	Control group (*n* = 34)	RE group (*n* = 40)	t/χ^2^/Z	*P*
Age (mean ± SD, years)	47.94 ± 8.09	47.88 ± 11.40	−0.029	0.977
Gender [*n* (%)]			2.874	0.092
Male	22 (64.71)	18 (55.00)		
Female	12 (35.29)	22 (45.00)		
Height (mean ± SD, cm)	167.79 ± 7.06	169.15 ± 8.59	0.734	0.465
Weight (mean ± SD, kg)	67.47 ± 8.27	65.44 ± 13.48	−0.794	0.430
BMI (mean ± SD, kg/m^2^)	23.99 ± 2.41	22.70 ± 3.41	−1.899	0.062
GERDQ [*M* (*Q_25_*, *Q_75_*)]	6.00 (6.00, 6.00)	9.50 (8.00, 12.00)	6.178	<0.001
Heartburn	0.00 (0.00, 0.00)	2.00 (1.00, 3.00)	5.309	<0.001
Regurgitation	0.00 (0.00, 0.00)	2.00 (1.00, 3.00)	6.806	<0.001
Epigastric pain	2.00 (1.00, 3.00)	3.00 (3.00, 3.00)	3.573	<0.001
Nausea	3.00 (2.00, 3.00)	3.00 (3.00, 3.00)	2.241	<0.001
Sleep disturbance	0.00 (0.00, 0.00)	0.00 (0.00, 2.00)	4.095	<0.001
Additional medication	0.00 (0.00, 0.00)	0.00 (0.00, 2.25)	4.413	<0.001
HADS-A [*M* (*Q_25_*, *Q_75_*)]	3.00 (3.00, 4.00)	8.00 (5.00, 11.75)	5.615	<0.001
HADS-D [*M* (*Q_25_*, *Q_75_*)]	3.00 (2.00, 4.25)	6.00 (3.00, 9.00)	3.59	<0.001

### Comparison of MTV between the two groups

**Figure 2:**
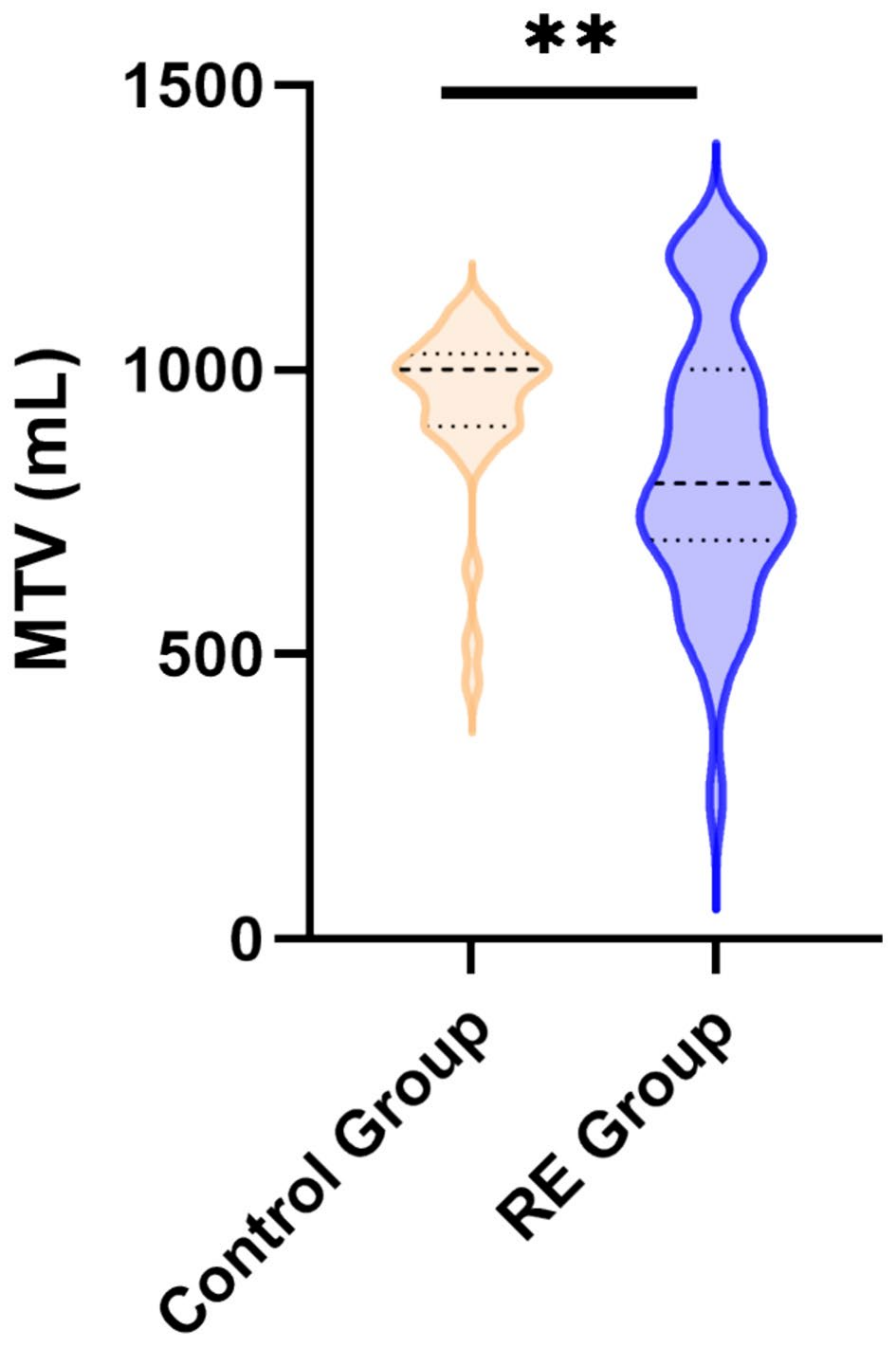
Compared to the control group, the MTV in the RE group was significantly lower (*p* < 0.01).

The MTV in the RE group [800.00 (700.00, 1000.00)] was significantly lower than that in the control group [1000.00 (900.00, 1050.00)] (*p* < 0.05) (Figure 2).

### Comparison of VAS scores between the two groups at different time points

**Figure 3:**
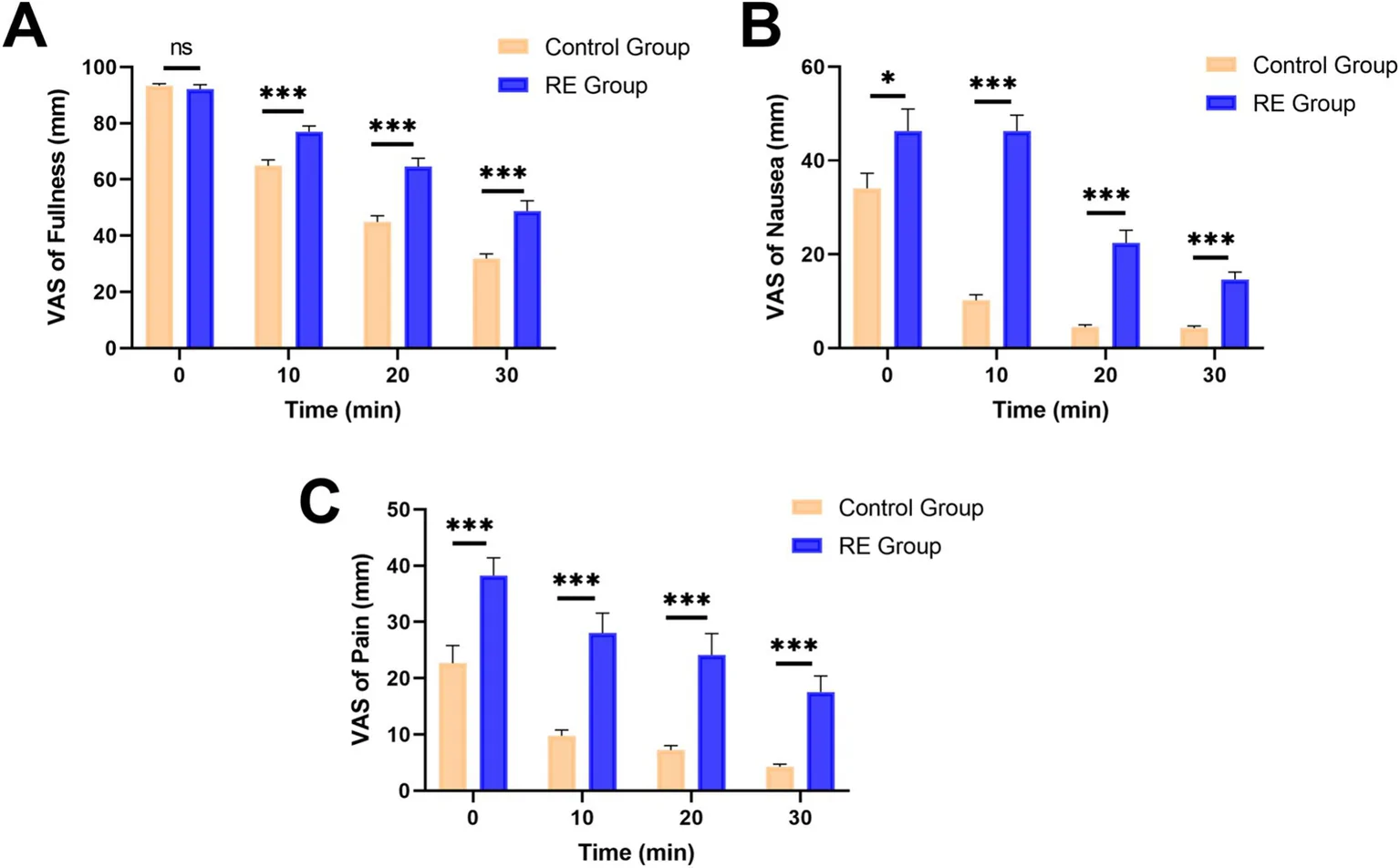
Comparison of VAS scores between the control group and RE group. **(A)** VAS of fullness; **(B)** VAS of nausea; **(C)** VAS of pain. *p < 0.05, ***p < 0.001.

Figure 3 illustrates the changes in VAS scores for fullness, nausea, and pain over time in both groups. No significant difference in fullness score was observed at baseline; however, the RE group had significantly higher scores at all subsequent time points (all *p* < 0.001), despite a gradual decline in both groups. The RE group also had a significantly higher nausea score at baseline (*p* < 0.05), and although it decreased over time, it remained significantly higher than that of the control group at each time point (all *p* < 0.001). Pain scores in the RE group were significantly higher at all postprandial time points (all *p* < 0.001), with scores decreasing gradually in both groups.

To account for the potential confounding by differential ingested volume, we calculated a symptom load index (VAS/MTV) to normalize postprandial symptom severity. After adjusting for the actual volume consumed, the RE group still exhibited a significantly higher symptom load index for fullness at all postprandial time points (all *p* < 0.01), for nausea at 10, 20, and 30 min (all *p* < 0.001), and for pain at all time points (all *p* < 0.001). Only the nausea symptom load index at 0 min did not differ significantly between the two groups (*p* = 0.160, Table 2).

**Table 2:** Symptom load index (VAS/MTV) between the control and RE group.

VAS/MTV		Control group (*n* = 34)	RE group (*n* = 40)	Z	*P*
Fullness	0 min	9.50 (9.00, 10.56)	11.94 (9.63, 14.05)	−2.666	0.008
	10 min	6.34 (5.65, 8.00)	10.00 (7.53, 11.61)	−4.151	<0.001
	20 min	4.21 (3.78, 5.14)	8.38 (6.08, 10.00)	−4.693	<0.001
	30 min	2.00 (1.58, 2.86)	6.37 (4.04, 8.43)	−5.083	<0.001
Nausea	0 min	3.79 (3.22, 4.79)	5.23 (2.23, 9.17)	−1.405	0.160
	10 min	1.05 (0.37, 1.66)	5.64 (2.79, 7.97)	−6.839	<0.001
	20 min	0.48 (0.20, 0.74)	2.05 (1.19, 3.89)	−6.634	<0.001
	30 min	0.32 (0.10, 0.50)	1.92 (0.54, 2.78)	−5.003	<0.001
Pain	0 min	2.00 (0.800, 3.43)	4.56 (2.50, 6.84)	−4.052	<0.001
	10 min	0.96 (0.49, 1.64)	2.50 (1.57, 4.40)	−4.757	<0.001
	20 min	0.79 (0.30, 1.14)	1.50 (0.93, 5.45)	−3.745	<0.001
	30 min	0.48 (0.22, 0.70)	1.52 (0.83, 2.70)	−4.624	<0.001

### Comparison of gastric emptying rate (GER%) between the two groups

**Figure 4:**
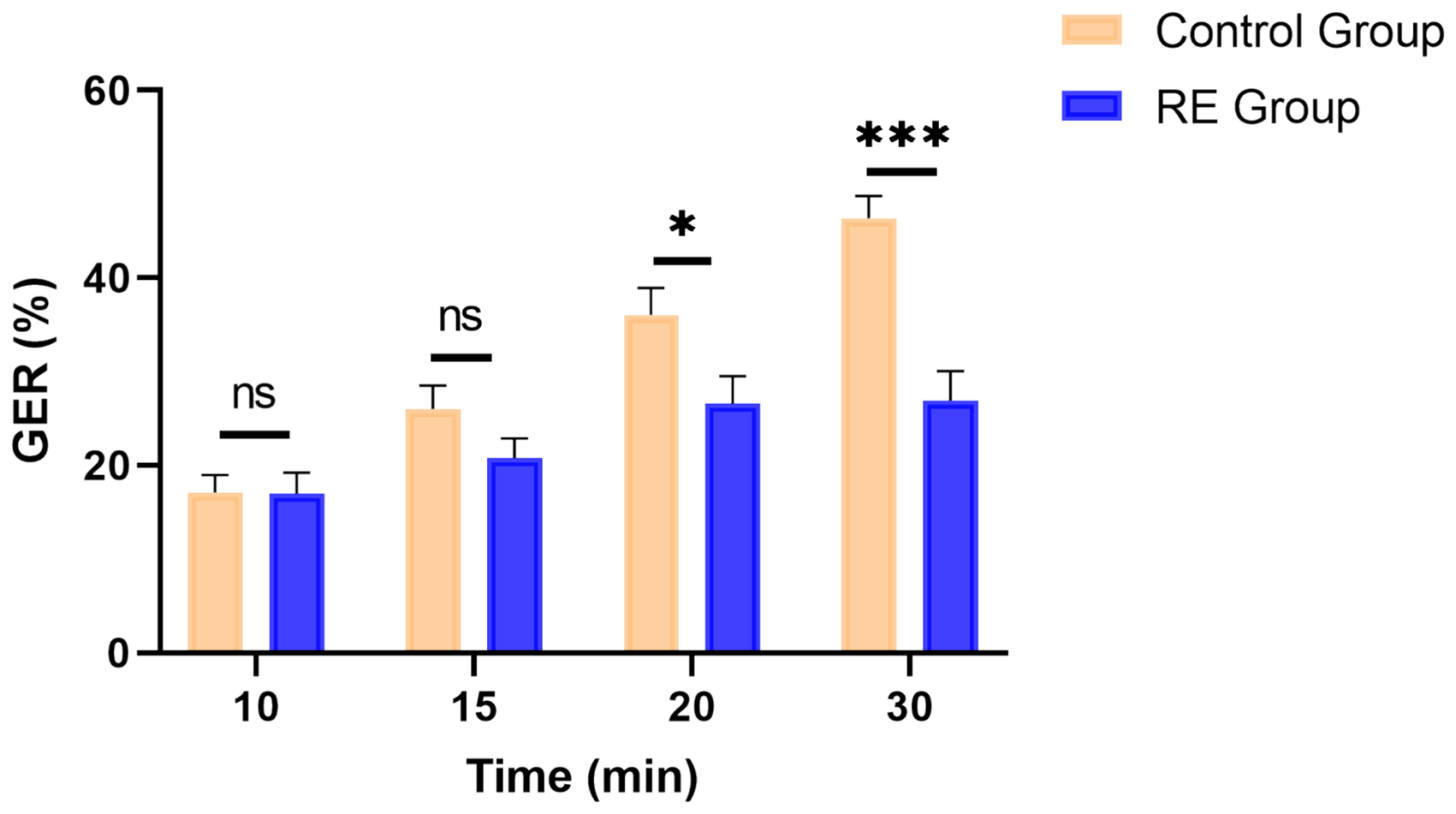
Comparison of gastric emptying rate (GER%) between the control group and RE group. *p < 0.05, ***p < 0.001.

There were no significant differences in GER% between the two groups at 10 min and 15 min, but the GER% in the RE group was significantly lower at 20 min and 30 min (*p* < 0.05) (Figure 4).

### Comparison of EGG results between the two groups

**Figure 5:**
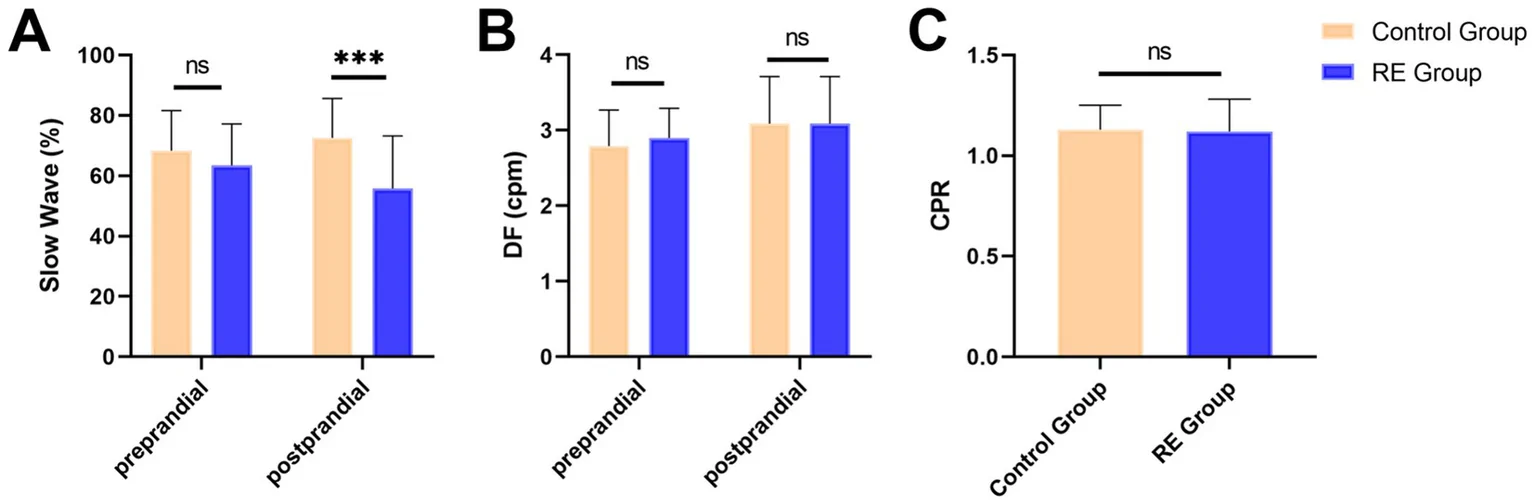
Comparison of EGG between the control group and RE group. **(A)** Comparison of normal gastric slow wave percentage (preprandial vs. postprandial). **(B)** Comparison of DF (preprandial vs. postprandial). **(C)** Comparison of the ratio of postprandial to preprandial DP. ***p < 0.001.

The EGG results showed that the proportion of normal postprandial slow waves in the RE group was significantly lower than that in the control group (*p* < 0.01). No statistically significant differences were found in DF or CPR between the two groups in either the preprandial or postprandial state (Figure 5).

### Correlation analysis between difference indicator and two main symptoms of GERDQ scale

Based on multidimensional assessments of gastric motility, the RE group found the following abnormal parameters, including MTV, 10-, 20- and 30-min VAS of fullness scores, 0–10, and 20-min VAS of nausea scores, 0–10, 20- and 30-min VAS of pain scores. In addition, 20 min GER%, 30 min GER% and postprandial slow wave ratio were abnormal.

Correlation analyses were performed within the RE group to explore associations between gastric motility parameters and the two most common symptoms, heartburn and regurgitation (Table 3). Heartburn showed a significant positive correlation only with pain score at 0 min (*r* = 0.442, *p* = 0.004). Regurgitation was significantly positively correlated with gastric emptying rate (GER%) at 30 min (*r* = 0.512, *p* = 0.001). No other significant correlations were observed between heartburn or regurgitation and any of the other parameters, including MTV, fullness scores at all time points, nausea scores, pain scores at other time points, postprandial slow-wave proportion, or GER% at 20 min (all *p* > 0.05).

**Table 3:** Correlation analysis between gastric motility parameters and main symptoms (heartburn and regurgitation) in the RE group.

Parameters		Heartburn		Regurgitation	
		*r*	*P*	*r*	*P*
MTV		−0.027	0.868	−0.050	0.706
Fullness	10 min	−0.040	0.806	0.137	0.398
	20 min	−0.201	0.213	0.076	0.642
	30 min	−0.269	0.094	0.147	0.367
Nausea	0 min	0.028	0.865	−0.248	0.122
	10 min	0.255	0.112	−0.070	0.668
	20 min	0.165	0.310	−0.063	0.700
Pain	0 min	0.442	0.004	−0.179	0.268
	10 min	0.201	0.213	−0.101	0.534
	20 min	0.186	0.251	0.017	0.915
	30 min	0.233	0.149	0.115	0.479
Slow wave	Postprandial	0.180	0.266	0.088	0.589
GER%	20 min	−0.180	0.267	0.272	0.089
	30 min	−0.150	0.354	0.512	0.001

### Gastrokinetic parameters in patients with RE

The resting pressure of HRM UES (U RP), the resting pressure of LES (L RP), the distal contraction integral (DCI), and the percentage of ineffective swallows (ISP) were selected as parameters reflecting esophageal dynamics, and the data analysis and the range of reference values of the RE group were shown in Table 4.

**Table 4:** Esophageal dynamic parameters in reflux esophagitis group.

Research data	RE group (*n* = 40)	Reference range
U RP [*M* (*Q_25_*, *Q_75_*), mmHg]	22.00 (14.78, 36.50)	34–104
L RP [*M* (*Q_25_*, *Q_75_*), mmHg]	7.50 (6.00, 11.75)	13–43
DCI [*M* (*Q_25_*, *Q_75_*), mmHg·s·cm]	847.90 (545.63, 1242.08)	450 ~ 8,000
ISP (%)	15.00 (0.00, 53.41)	<30

### Correlation analysis between gastric dynamic parameters and esophageal dynamic parameters

**Figure 6:**
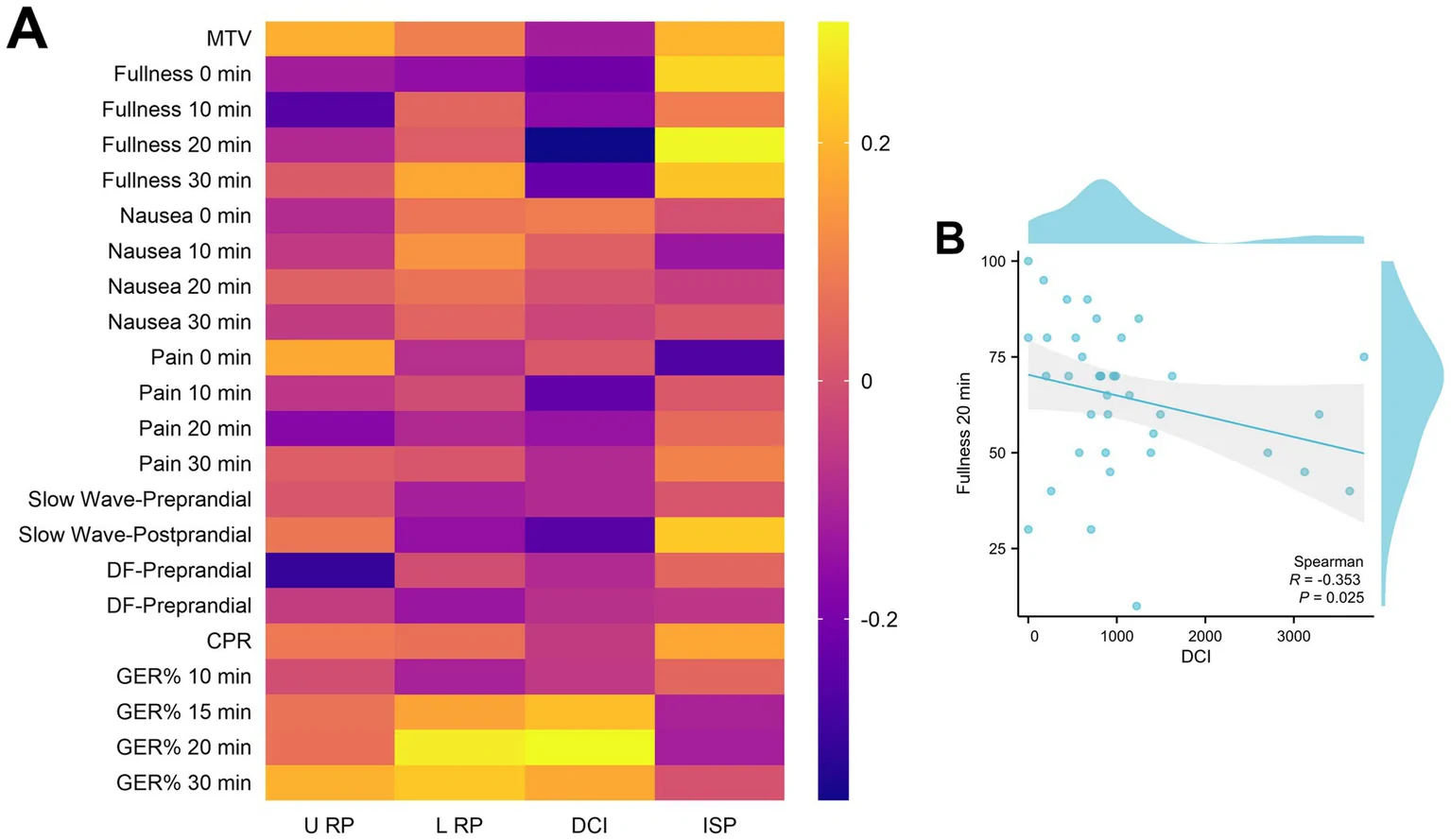
Correlation analysis between gastric motility parameters and esophageal motility parameters in patients with RE. **(A)** Heatmap showing the correlation matrix between gastric indices and esophageal parameters. Color intensity represents the correlation coefficient (*r*). **(B)** Scatter plot illustrating the negative correlation between postprandial fullness score at 20 min and DCI.

The correlations between gastric motility indices (including MTV, fullness, nausea, pain scores at different time points, slow-wave parameters, DF, CPR, and GER% at various time points) and esophageal motility parameters (UES resting pressure, LES resting pressure, DCI) were examined using Spearman/Pearson correlation analysis (Table 5; Figure 6A). As shown in Figure 6B, only the fullness score at 20 min postprandial was significantly negatively correlated with the DCI in the RE group (*r* = −0.353, *p* = 0.025). No other significant correlations were observed between any gastric parameters and esophageal motility indices (all *p* > 0.05).

**Table 5:** Correlation analysis between gastric motility parameters and esophageal motility parameters in patients with RE.

Parameters		U RP		L RP		DCI		ISP	
		*r*	*P*	*r*	*P*	*r*	*P*	*r*	*P*
MTV		0.186	0.25	0.096	0.554	−0.122	0.455	0.193	0.232
Fullness	0 min	−0.124	0.446	−0.157	0.334	−0.213	0.188	0.249	0.121
	10 min	−0.256	0.111	0.045	0.783	−0.167	0.304	0.092	0.573
	20 min	−0.096	0.555	0.024	0.883	−0.353	0.025	0.299	0.061
	30 min	0.021	0.896	0.172	0.29	−0.228	0.158	0.221	0.17
Nausea	0 min	−0.090	0.581	0.075	0.645	0.089	0.585	−0.006	0.969
	10 min	−0.056	0.731	0.134	0.409	0.030	0.853	−0.138	0.395
	20 min	0.032	0.845	0.071	0.665	0.000	1.000	−0.046	0.776
	30 min	−0.055	0.735	0.038	0.818	−0.030	0.855	0.010	0.95
Pain	0 min	0.174	0.284	−0.081	0.617	0.013	0.936	−0.266	0.097
	10 min	−0.067	0.679	−0.015	0.928	−0.233	0.148	0.012	0.944
	20 min	−0.174	0.282	−0.096	0.554	−0.146	0.37	0.052	0.748
	30 min	0.027	0.867	0.007	0.968	−0.092	0.574	0.102	0.531
Slow wave	Preprandial	0.008	0.961	−0.118	0.468	−0.093	0.569	0.008	0.961
	Postprandial	0.078	0.634	−0.152	0.349	−0.254	0.114	0.228	0.158
DF	Preprandial	−0.306	0.055	−0.011	0.949	−0.091	0.579	0.045	0.782
	Postprandial	−0.052	0.748	−0.138	0.396	−0.078	0.634	−0.067	0.681
CPR		0.082	0.613	0.067	0.681	−0.055	0.734	0.171	0.292
GER%	10 min	−0.011	0.948	−0.113	0.488	−0.059	0.716	0.045	0.781
	15 min	0.071	0.664	0.164	0.311	0.210	0.194	−0.111	0.496
	20 min	0.067	0.683	0.283	0.077	0.302	0.058	−0.118	0.468
	30 min	0.189	0.242	0.225	0.163	0.174	0.284	0.000	1.000

## Discussion

This study systematically evaluated the gastric motility function in patients with RE. Compared with the healthy control group, patients with RE showed significantly reduced gastric tolerance, enhanced visceral sensitivity, delayed gastric emptying, and weakened postprandial gastric slow-wave activity. Moreover, several gastric parameters were correlated with typical RE symptoms and esophageal motility disorder indicators, confirming the pathological physiological connection between gastric dysfunction in RE and esophageal dysfunction. These results suggest that RE may be a disorder of upper gastrointestinal motility.

It is commonly acknowledged that one of the pathophysiological causes driving GERD is gastric motility. The GERDQ is a widely utilized diagnostic instrument, and prior research has demonstrated a good correlation between GERDQ scores and the endoscopic severity of RE (21). This study found that GERDQ scores were significantly higher in the RE group than in healthy controls. Among the individual items, heartburn and regurgitation showed the most prominent increases, indicating that these two symptoms are the most common clinical manifestations in RE patients which is consistent with established knowledge (22).

The ability of the proximal stomach to relax in reaction to a meal, enabling an increase in gastric volume without a notable rise in intragastric pressure, is known as gastric accommodation. The MTV during a nutrient drink test in the current investigation was considerably lower in RE patients than in healthy controls, indicating reduced gastric tolerance. While MTV is a widely used with reported sensitivity of 92.0% and specificity of 86.0% for identifying inadequate accommodation in functional dyspepsia (23). It is important to recognize that MTV reflects a composite of accommodation, gastric wall tension, and visceral sensitivity, rather than accommodation alone. In our investigation, RE patients demonstrated both heightened visceral sensitivity and reduced tolerance to gastric distension, a pattern similar to that observed in functional dyspepsia (FD) patients. VAS is commonly used to assess symptoms such as pain and nausea. In this study, compared to the control group, the total amount of food intake by RE patients was lower, but the VAS scores for fullness, nausea, and pain at all post-meal time points were significantly higher. This indicates that RE patients have high sensitivity of the gastric organ. It is important to note that VAS scores and MTV were determined during the same nutrient challenge. Because patients with lower MTV consumed smaller volumes, they experienced less gastric distension, which could theoretically confound the interpretation of symptom severity. However, when we normalized VAS scores to the ingested volume (symptom load index), RE patients still demonstrated a significantly higher symptom load for fullness, pain, and nausea (except at 0 min) at all postprandial time points, supporting that the observed symptom differences reflect genuine visceral hypersensitivity rather than merely differential mechanical distension. This phenomenon has been widely recognized in functional dyspepsia, but there have been few studies on RE (24). Existing evidence suggests that these two abnormalities may share a common neurophysiological regulatory pathway via the brain-gut axis. Mechanical distension of the proximal stomach triggers afferent vagal signals that are transmitted to the nucleus tractus solitarius in the brainstem and subsequently relayed to higher brain regions involved in central pain modulation and emotional processing. In patients with functional gastrointestinal disorders, central sensitization has been documented, leading to abnormally amplified processing of visceral afferent signals, which in turn gives rise to visceral hypersensitivity and impaired gastric accommodation. In the present study, the significantly elevated scores for anxiety and depression further indicate that psychological factors can modulate gastric sensory and motor functions. A prospective cohort study has confirmed that anxiety symptoms are independently associated with reflux symptoms in gastroesophageal reflux disease (GERD), with anxious individuals being more susceptible to developing the disease and experiencing more severe symptoms (25). Moreover, a multicenter cross-sectional study reported that patients with refractory GERD had significantly higher anxiety and depression scores than those with non-refractory GERD, suggesting that negative emotional states may reduce the therapeutic response to proton pump inhibitor (PPI) therapy (26). In summary, patients with reflux esophagitis commonly exhibit anxiety and depression in addition to typical reflux symptoms. This finding indicates that enhanced perception of gastric distension-related signals may lower the symptom perception threshold in RE patients.

Gastrointestinal contrast-enhanced ultrasound is a non-invasive imaging technique that uses orally administered contrast agents to evaluate gastric emptying without radiation exposure, offering advantages in safety, repeatability, and real-time dynamic observation compared to radionuclide scintigraphy (27, 28). The results of this study showed that RE patients showed significantly lower gastric emptying rates at 20 and 30 min postprandially, indicating delayed gastric emptying. This finding is consistent with the positive correlation between gastropathy and esophageal motility disorders reported by Zikos et al. (29). In the present study, regurgitation severity was positively correlated with the 30-min gastric emptying rate. Although this result appears to contrast with the common expectation that delayed emptying aggravates reflux, it should be interpreted with caution, as the correlation does not imply causation. This paradoxical phenomenon may be explained by a combination of multiple mechanisms. We used an ultrasound contrast agent to assess liquid-phase emptying, a method that may not accurately reflect solid-phase emptying dynamics. During the early postprandial period, rapid emptying of liquid meals may lead to acute gastric distension and transient lower esophageal sphincter relaxation, thereby increasing the risk of reflux. In addition, severe regurgitation may cause gastric contents to be lost through the esophagus, paradoxically reducing residual gastric volume and accelerating the emptying rate as visualized on ultrasound. Meanwhile, intragastric pressure dysregulation in patients with severe regurgitation may simultaneously affect symptomatic presentation and the propulsive emptying of liquids. Future studies combining high-resolution manometry with gastric emptying imaging are necessary to elucidate these complex interrelationships.

It is currently widely accepted that the pacemaker potential of gastric contraction is the slow wave, which not only forms the basis of gastric myoelectrical activity but also governs gastric contractile movements (30). The frequency of myoelectrical activity measured by surface electrogastrography is highly consistent with that recorded *in vivo* (31, 32). Therefore, this study used surface electrogastrography to evaluate gastric myoelectrical function in patients. The results showed that the percentage of normal postprandial slow waves was significantly reduced in patients with reflux esophagitis, indicating the presence of gastric myoelectrical abnormality. This finding is consistent with the meta-analysis which also found that patients with GERD had significantly less normal gastric slow-wave activity than healthy controls (31). However, no significant changes in the dominant frequency or dominant power of gastric myoelectrical activity in patients, indicating that the pathological process primarily disrupts the stability of gastric slow-wave rhythm rather than affecting the intrinsic frequency or amplitude of the pacemaker potential. The characteristic of this pattern is a decrease in the proportion of normal slow waves, while the dominant frequency and power remain unchanged, similar to the situation observed in functional dyspepsia. This indicates that similar electrophysiological abnormalities may exist in upper gastrointestinal functional diseases. The cellular origin of gastric rhythm disorders is mainly attributed to the dysfunction of interstitial cells of Cajal (ICC). These cells act as pacemakers of the stomach and are responsible for generating gastric slow waves and regulating smooth muscle contraction through gap junctions (33). Studies have shown that there is a reduction in the number of ICC or impaired function in gastroparesis and functional dyspepsia (34, 35). In this study, the decrease in the normal proportion of gastric slow waves after meals in patients may reflect the impaired stability of the ICC network, which in turn affects the contraction of the antrum and the coordination function of the pylorus, possibly leading to delayed gastric emptying and may be associated with an increased risk of transient lower esophageal sphincter relaxation.

The correlation analysis between heartburn and reflux in RE patients and abnormal gastric motility parameters indicated that the score of satiety 20 min after a meal was negatively correlated with DCI. This suggests that patients with higher visceral sensitivity or more impaired gastric tolerance may have weaker esophageal body contraction ability. This is consistent with the results of previous high-resolution manometry (HRM) studies, which found a direct correlation between esophageal motility dysfunction, dysfunction of the esophagogastric junction, and reflux load (36). Another study has shown that high-grade RE is associated with weakened esophageal peristalsis and decreased pressure in the lower esophageal sphincter (37). Zikos et al. (29) demonstrated that abnormal gastric emptying imaging (GES) is positively correlated with HRM functional abnormalities, which supports the hypothesis that gastric motility disorders and esophageal motility disorders share a common pathogenesis. Similarly, Triadafilopoulos et al. (38) reported that patients with delayed gastric emptying symptoms exhibited a higher prevalence of esophageal motility disorders. However, this study did not observe any correlation between the gastric emptying parameters or slow wave parameters of RE patients and the resting pressure of the lower esophageal sphincter. This suggests that the gastric dysfunction in RE patients may not be solely secondary to the dysfunction of the lower esophageal sphincter. It is important to note that we did not classify the RE cohort as having generalized “esophageal hypomotility” based on any single parameter. While the median ineffective swallow percentage (ISP) of 15% fell within the normal range defined by the Chicago Classification v3.0 (<30%), the broad interquartile range (0.00–53.41%) indicated substantial inter-individual variability, with a subset of patients exhibiting values above the 30% threshold. Moreover, the significantly reduced LES resting pressure (7.50 mmHg) and the negative correlation between gastric fullness and DCI collectively support the presence of selective esophageal motor abnormalities in this cohort, rather than global hypomotility.

However, this study has several limitations. The sample size is small and the study is single-center, which may limit generalizability. The MTV from the nutrient drink test is an indirect measure that reflects a composite of gastric accommodation, wall tension, and visceral sensitivity, rather than accommodation alone; true gastric accommodation is best assessed by the barostat or MRI. Additionally, although VAS scores were measured after the same nutrient challenge used for MTV, normalization to ingested volume (symptom load index) supported that the elevated symptom perception is not solely attributable to reduced gastric distension; nevertheless, a separate fixed-volume challenge would provide a more rigorous independent assessment. This study applied the Chicago Classification v3.0 for HRM interpretation, as data were collected prior to the 2021 publication of v4.0; however, our analysis relied on continuous HRM parameters rather than categorical diagnoses. Besides, gastric emptying was assessed by ultrasound measurement of antral area reduction, which reflects liquid-phase emptying rather than whole-stomach or solid-phase emptying, and its validity is less established than scintigraphy. Although all gastric and esophageal function assessments were conducted by a single trained investigator to minimize interobserver variability; while this ensures internal consistency, it precludes the estimation of interobserver reliability. Finally, psychological confounders such as anxiety and depression were not adjusted for in the primary analyses. The elevated HADS scores in the RE group may have independently influenced gastric tolerance, visceral sensitivity, and EGG parameters via the brain-gut axis.

## Conclusion

In summary, this study evaluated the gastric motility and esophageal function of patients with RE, and found that the gastric pathophysiological abnormalities in RE patients included reduced gastric tolerance, increased visceral sensitivity, delayed gastric emptying, and gastric rhythm disorders. These results indicate that gastric motility plays an important role in the pathogenesis of RE. Moreover, gastric motility disorders may be related to selective esophageal motor abnormalities, including impaired LES barrier function and reduced esophageal body contractile vigor, suggesting that gastric function and esophageal function should not be viewed in isolation. Therefore, assessment of gastric function is particularly important in patients with refractory or atypical RE.

## Data Availability

The raw data supporting the conclusions of this article will be made available by the authors, without undue reservation.
